# Facile Zn and Ni Co-Doped Hematite Nanorods for Efficient Photocatalytic Water Oxidation

**DOI:** 10.3390/nano12172961

**Published:** 2022-08-27

**Authors:** Joan Talibawo, Pannan I. Kyesmen, Marie C. Cyulinyana, Mmantsae Diale

**Affiliations:** 1 African Centre of Excellence in Energy and Sustainable Development, University of Rwanda, KN 67 Street Nyarugenge, P.O. Box 3900, Kigali 4285, Rwanda; 2 Department of Physics, University of Pretoria, Private Bag X20, Hatfield 0028, South Africa

**Keywords:** hematite nanorods, zinc/nickel co-doping, photocurrent, PEC water oxidation

## Abstract

In this work, we report the effect of zinc (Zn) and nickel (Ni) co-doping of hydrothermally synthesized hematite nanorods prepared on fluorine-doped tin oxide (FTO) substrates for enhanced photoelectrochemical (PEC) water splitting. Seeded hematite nanorods (NRs) were facilely doped with a fixed concentration of 3 mM Zn and varied concentrations of 0, 3, 5, 7, and 9 mM Ni. The samples were observed to have a largely uniform morphology of vertically aligned NRs with slight inclinations. The samples showed high photon absorption within the visible spectrum due to their bandgaps, which ranged between 1.9–2.2 eV. The highest photocurrent density of 0.072 mA/cm^2^ at 1.5 V vs. a reversible hydrogen electrode (RHE) was realized for the 3 mM Zn/7 mM Ni NRs sample. This photocurrent was 279% higher compared to the value observed for pristine hematite NRs. The Mott–Schottky results reveal an increase in donor density values with increasing Ni dopant concentration. The 3 mM Zn/7 mM Ni NRs sample produced the highest donor concentration of 2.89 × 10^19^ (cm^−3^), which was 2.1 times higher than that of pristine hematite. This work demonstrates the role of Zn and Ni co-dopants in enhancing the photocatalytic water oxidation of hematite nanorods for the generation of hydrogen.

## 1. Introduction

Solar water splitting is one of the promising technologies for the green and sustainable production of hydrogen, a clean chemical energy carrier with a relatively high gravimetric energy density of 143 MJ kg^−1^ [1]. The technology uses photoactive electrodes for photon absorption in a PEC cell setup that also comprises an aqueous electrolyte, a reference electrode, and a counter electrode usually made of platinum. During photocatalysis, when the photoelectrode absorbs photons with energy equivalent to or more than its bandgap, electron–hole (e^−^/H^+^) pairs are generated in the electrode. If the photoelectrode is an n-type semiconductor, the photogenerated electrons are excited from the valence band into the conduction band, leaving behind holes. The electrons move through an external circuit to the counter electrode, whereas the holes shift to the photoelectrode–electrolyte interface. This results in the oxidation of water at the photoanode and, consequently, the reduction of H^+^, leading to the evolution of hydrogen at the counter electrode of the PEC device [2,3]. An effective photoelectrode material is expected to have a favorable bandgap that allows for high photon absorption in the visible spectrum to enable sufficient photogeneration of the charge carriers required to perform photo-induced redox reactions for hydrogen production [4,5].

Therefore, when choosing a photoelectrode material for PEC applications, it is important to carefully consider the key optical and other catalytic properties of the photocatalyst [6,7]. In addition, the earth abundance of the material is also critical for the sustainability of hydrogen production via PEC reactions [8]. Hematite is one of the highly investigated metal oxides for use as photoanode material for PEC reactions because of its favorable bandgap (1.9–2.1 eV), which is capable of absorbing ~40% of photons in the visible region [9], abundance in nature, stability in a broad pH range, and non-toxicity [10]. However, the PEC performance of hematite is greatly hampered by the ultrafast charge recombination in its bulk and surface because of its short hole-diffusion path of 2–20 nm, poor electrical conductivity, and slow PEC kinetics for oxygen evolution, among others [11]. In a bid to enhance the performance of hematite electrodes, a range of strategies has been studied, such as using heterostructures [12,13], nanoscale engineering [14], seed layers [15], and elemental doping [16].

Nanoscale engineering of semiconductor materials used in PEC water splitting has been outstanding among other strategies [17]. With this strategy, nanostructures such as nanosheets [18], nanoparticles [19], and nanorods (NRs) [20] are among the widely investigated structures for PEC applications because of their explicit and unique potential in modifying the properties of hematite for enhanced photon absorption and charge transport. NRs are one of the promising nanostructures investigated for PEC water splitting because they provide a wider surface area for photocatalytic reactions [21,22]. NRs have also been reported to exhibit better e^−^/H^+^ mobility than nanospheres [9]. Moreover, when NR films are subjected to high-temperature annealing for a short time, their crystallinity is improved, which directly enhances charge carrier transport during PEC reactions [23]. Although several techniques, such as electrochemical deposition [24], pulsed laser deposition (PLD) [25], and spray pyrolysis [26], have been used for the preparation of various hematite electrodes, the hydrothermal technique is considered to be flexible, simple, and inexpensive for the fabrication of NRs [27,28]. 

The use of seed layers between substrates and a semiconductor photoanode has been reported to enhance their photocurrent outputs through the suppression of charge recombination at the substrate–semiconductor interface [29]. For example, it has been demonstrated that synthesizing hematite film over tungsten trioxide (WO_3_)-seeded FTO increased its photocurrent density by 125% [30]. Relatedly, zinc (Zn)-seeded substrates have been reported to enhance the nucleation and growth of vertically oriented Zn NRs [31,32]. However, not much has been explored on the PEC properties of hematite NRs grown on hematite-seeded FTO substrates. In addition, the use of seed layers comprising different elements from those of the NRs could induce doping at relatively elevated temperatures from the ion species of the seed layer [33]. It is therefore important to use the same material element for the NRs film and maintain the seed layer in controlled conditions to avoid indirect doping. Furthermore, elemental doping of the photoelectrodes is another approach to improve the electrical conductivity and photon absorption of hematite nanostructures [27,34]. In addition, doping has been reported to suppress the recombination of e^−^/H^+^ pairs and consequently improve the photocurrent densities of hematite NRs [35]. The influence of various metal and non-metal dopants, such as Sn [36], sulfur (S) [37], and silver (Ag) [3], has been investigated. For example, a comparative study by Jinzhan et al. (2016) on the effect of different metal atom dopants on the PEC performance of hydrothermally synthesized hematite NRs demonstrated improved photocurrent outputs with titanium (Ti), zirconium (Zr), and Sn dopants [38]. Doping hematite films with Zn in work conducted by Aadesh et al. (2017) doubled the photocurrent density of the bare films to 0.81 mA/cm^2^ at 1.23 V vs. RHE as a result of enhanced charge transport [10]. Moreover, Zn has been reported to be among the best dopants for enhancing the photocurrent density of hematite films [3,26]. In addition, investigations on Ni doping of hematite NRs resulted in a 1.3-fold improvement in photocurrent density compared to that of the undoped NRs, which was further doubled to 1.28 mA/cm^2^ by co-doping with cobalt (Co) [39]. The review by Shengnan et al. also demonstrates that facile doping of hematite with a suitable element enhances charge separation and transport dynamics, which consequently boosts its PEC performance [40]. However, despite the numerous research studies conducted on the effect of different dopants on the PEC performance of hematite NRs, no work has been reported on the effect of Ni and Zn as surface co-dopants of hematite towards improving the semiconductor photocatalytic properties. 

In this work, hydrothermally synthesized hematite NRs were co-doped with Zn and Ni to enhance their PEC performance. First, 3 mM Zn and 3, 5, 7, and 9 mM Ni dopants were spin-coated over pristine hematite NRs to obtain the Zn and Zn/Ni co-doped NR samples. The 3 mM Zn/7 mM Ni NRs samples exhibited the highest photocurrent of 72 mA/cm^2^ at 1.5 V vs. RHE due to the high donor density 2.89 × 10^19^ (cm^−3^) observed for the films. Based on the known literature, no work has been reported on the effect of facile Zn and Ni co-doping of hematite NRs that were hydrothermally grown over a hematite seed layer for the enhancement of PEC performance.

## 2. Materials and Methods

### 2.1. Materials and Substrate Preparation

Ferric chloride hexahydrate (FeCl_3_·6H_2_O), sodium nitrate (NaNO_3_), zinc nitrate hexahydrate (Zn(NO_3_)_2_·6H_2_O), and nickel nitrate hexahydrate (Ni(NO_3_)_2_·6H_2_O) were obtained from Sigma-Aldrich, South Africa, and used as received. The FTO substrates were first cleaned in an ultrasonic bath using sodium stearate (C_18_H_35_NaO_2_) soap solution, followed by deionized water (DI), ethanol, and finally acetone for ten minutes each. The substrates were then air-dried using nitrogen gas.

### 2.2. Experimental Procedure

**Figure 1:**
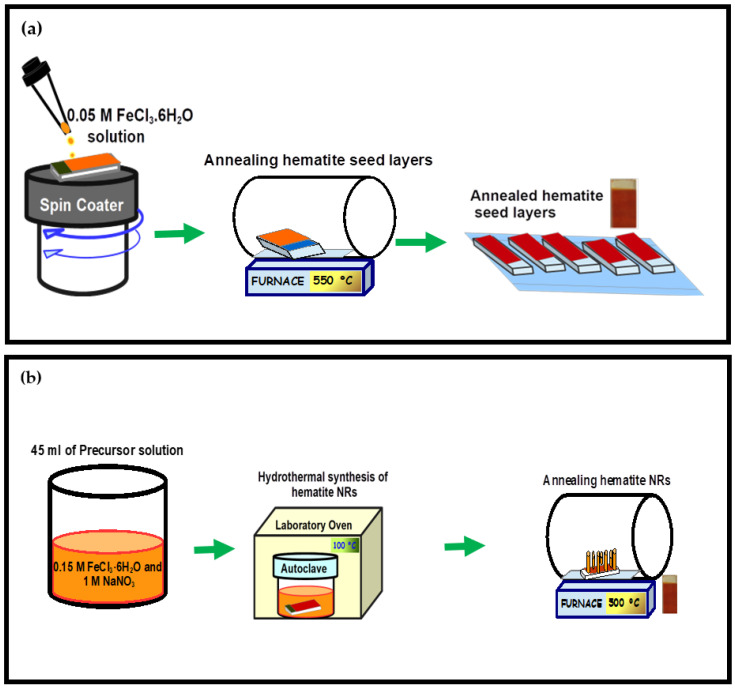
(**a**) Schematic illustration of the preparation of hematite seed layers on FTO by spin coating and (**b**) hydrothermal synthesis of hematite NRs.

Hematite seed layers were grown over the FTO substrates by spin coating a precursor solution of 0.05 M FeCl_3_.6H_2_O in ethanol at 5000 rpm. The process was repeated 8 times to grow the desired thickness of the seed layers, with 5 min intervals of drying at 70 °C in a laboratory oven. The hematite seed layers were then annealed at 550 °C for 1 h, followed by gradual cooling to room temperature. Figure 1a illustrates the experimental procedures for the preparation of the hematite seed layers on FTO substrates via spin coating.

The FTO substrates with the deposited hematite seed layers were placed at the bottom of an autoclave filled with 45 mL of an aqueous solution of thoroughly mixed 0.15 M FeCl_3_·6H_2_O and 1 M NaNO_3_, with the conducting side facing upwards. The autoclave was sealed and placed in a laboratory oven set to a temperature of 100 °C for 8 h to grow yellowish films of FeOOH on FTO. The deposited films were rinsed several times with DI water to remove the extra reactants and dried thereafter in a laboratory oven at 70 °C for 10 min. The FeOOH layers were then annealed at 500 °C for 2 h to obtain pristine hematite NRs (Figure 1b). 

**Figure 2:**
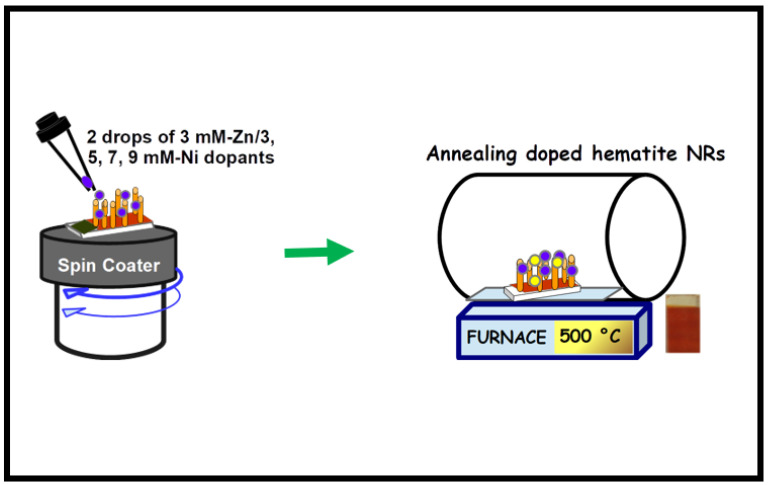
Illustration of Zn/Ni facile co-doping of hematite NRs.

Dopant concentrations of 3, 5, 7, and 9 mM were prepared from Ni(NO_3_)_2_·6H_2_O using ethanol as a solvent to serve as the Ni ion source. In addition, a 3 mM solution of Zn(NO_3_)_2_·6H_2_O was dissolved in ethanol and used as the Zn co-dopant ion source. Based on previous results, a 5–15% atomic composition of facile zinc dopant transformed the conventional n-type hematite film into a p-type photoanode [41]. This study selected an arbitrary but constant low dopant concentration of 3 mM Zn in order to preserve the n-type nature of the prepared hematite films. Two drops of the Zn dopant solution were spin-coated over the annealed hematite NR films for 30 s at 5000 rpm. The films were then dried in an oven at 70 °C for 10 min and subsequently annealed at 550 °C for 1 h to obtain the 3 mM Zn-doped NR samples. Furthermore, the 3 mM Zn-doped hematite NR samples were also spin-coated with two drops of 3, 5, 7, and 9 mM Ni dopant solutions, dried, and annealed in the same manner as the Zn-doped hematite films to obtain the 3 mM Zn/3 mM Ni, 3 mM Zn/5 mM Ni, 3 mM Zn/7 mM Ni, and 3 mM Zn/9 mM Ni NR co-doped samples, respectively. To avoid contamination of the samples, the pristine hematite NRs and the doped samples were annealed in separate tubes. Figure 2 shows the schematic representation of the doping and annealing process of the hematite NRs.

### 2.3. Characterization

The morphology of the as-prepared samples was examined using a Zeiss Ultra PLUS field-emission scanning electron (FESEM) microscope at 2 kV. Elemental composition analysis of the pristine and doped hematite NRs was performed using a Zeiss Crossbeam, which was connected to an energy-dispersive X-ray spectroscopy (EDS) system. The structural properties of the samples were investigated using a Bruker D2 Phaser X-ray diffractometer and a WITec alpha 300 RAS+ confocal micro-Raman microscope. An Agilent Cary-60 UV-vis spectrophotometer was used to measure the light absorption of the NRs within a 200–800 nm wavelength range.

PEC measurements were obtained using a VersaSTAT 3F potentiostat from Princeton Applied Research in a three-electrode cell with 1 M sodium hydroxide (NaOH) electrolyte, a 2 × 2 cm platinum (Pt) wire mesh as the counter electrode, and a silver/silver chloride (Ag/AgCl) reference electrode. A Newport Oriel, LCS-100™ solar simulator, calibrated to 1 sun at 100 mW/cm^2^, was used as the light source. The light was directed at the quartz window of the PEC cell, which allowed the illumination of a 0.49 cm^2^ surface area of the pristine and doped hematite photoelectrodes. Linear sweep voltammetry (LSV) measurements in the dark and under illumination were taken to study the current–voltage characteristics of the samples. All potential values were translated to the RHE scale according to Equation (1) [10].

(1) $$V_{RHE}=V_{Ag/AgCl}+\left(0.059pH\right)+V_{Ag/AgCl}^{0}$$

where $V_{RHE}$ represents potential in the RHE, $V_{\;}{}_{Ag/AgCl}^{0}$ = 0.1976 V at a standard temperature of 25 °C, $V_{Ag/AgCl\;}$ is the potential obtained from the experimental measurements using Ag/AgCl as the reference electrode, and the pH of the electrolyte is 13.6. The photocurrent measurement for pristine hematite NRs was used as the baseline result in the analysis of the photoresponse of the Zn and Zn/Ni co-doped samples. Mott–Schottky studies were conducted on the pristine and doped hematite samples in the dark at a constant frequency of 10,000 Hz, an AC amplitude potential of 10 mV, and a DC potential range of −1.2 to 0.5 V vs. Ag/AgCl. Electrochemical impedance measurements were conducted on illuminated photoanode samples using a 10 mV potential amplitude within a frequency range of 10,000 to 0.1 Hz at 0.23 V vs. Ag/AgCl.

## 3. Results

### 3.1. NR Morphology and Seed Layer Thickness

**Figure 3:**
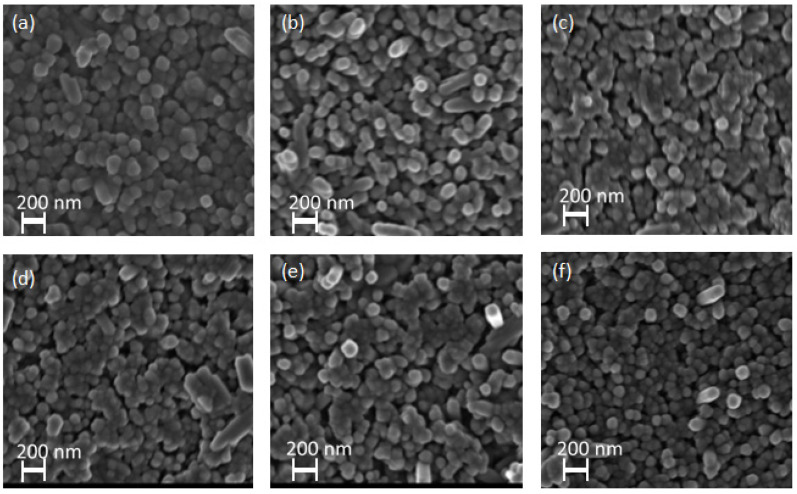
SEM images of (**a**) pristine hematite NRs, (**b**) 3 mM Zn-doped NRs, and (**c**) 3 mM Zn/3 mM Ni, (**d**) 3 mM Zn/5 mM Ni, (**e**) 3 mM Zn/7 mM Ni, and (**f**) 3 mM Zn/9 mM Ni co-doped NRs.

The surface morphologies of the pristine, Zn-doped, and Zn/Ni co-doped hematite NRs are shown in Figure 3. The NRs were generally closely packed in clusters and vertically aligned over the hematite seed layers. All samples exhibited similar morphology despite the variation in Ni dopant concentrations. This suggests that the surface morphology of the samples was only influenced by the hydrothermal reaction [42]. Similarly, studies by Feng Cheng et al. and other researchers confirmed that facile doping of hematite NRs with Ni had no effect on their surface morphology [39,43]. This was attributed to low Ni dopant concentrations loaded over the hematite NRs that limited the formation of particles and eventual agglomeration over the hematite films [44]. 

An average film thickness of ~127 nm was obtained for the seed layers using a profilometer alpha step. 

### 3.2. XRD Structural Characterization

**Figure 4:**
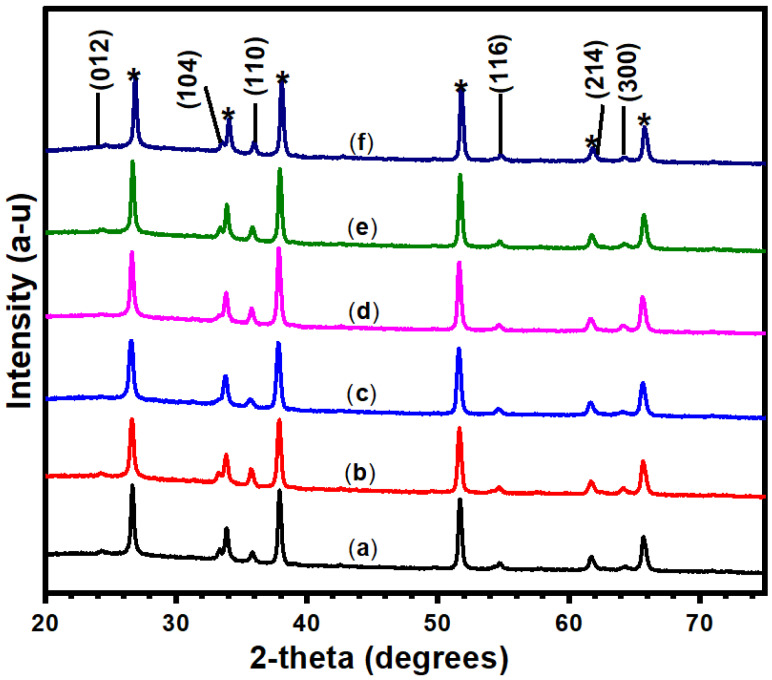
X-ray diffraction patterns of (**a**) pristine hematite NRs, (**b**) 3 mM Zn/3 mM Ni, (**c**) 3 mM Zn/5 mM Ni, (**d**) 3 mM Zn/7 mM Ni, (**e**) 3 mM Zn/9 mM Ni, and (**f**) 3 mM Zn-doped NRs.

To investigate the phase of the prepared pristine and doped hematite NRs, the XRD diffraction patterns of the samples given in Figure 4 were analyzed. The XRD results show the rhombohedral crystalline structure of hematite indexed to (104), (110), (116), (214), (300), and (125) planes with 2-theta peaks at 33.15°, 35.61°, 54.09°, 62.45°, 63.99°, and 66.03°, respectively, in line with XPert high score plus software analysis for the samples (reference code: 00-033-0664). The peaks correlate to the R-3C (167) space group with uniform lattice parameters of 0.50356 nm for *a* and *b* and 0.1374 nm for *c*. The peaks at (104), (110), (214), and (300) planes also match the rhombohedra crystalline phases of pristine and doped hematite NRs according to JCPDS card no. 24-0072 [38]. All samples show comparable intensities for the peaks in the (110) plane. It is in this plane that higher preferential charge separation and transport for PEC water splitting is reported [28,45]. The similar intensities observed across the peaks could be attributed to the uniform annealing temperature used during sample preparation, which can directly impact the sample’s crystallinity and charge mobility [46]. There were no observable peak shifts across the XRD patterns, in agreement with earlier reports [38]. This observation is an indication that the dopants had no significant impact on the structure of the hematite NRs [38,39]. The peaks did not show phases of Ni and Zn, implying that the dopants were incorporated into the hematite lattice and had no effect on the hematite crystal structure [28,47]. We attribute the successful incorporation of dopants within the crystal structure of hematite to the small concentrations and volume of Zn and Ni used during sample preparation [48]. In addition, Ni, Zn, and Fe have similar chemical properties since they belong to the same group of transition metals and the fourth period of the periodic table [39]. The intensity of the 3 mM Zn/5 mM Ni NR sample peaks was observed to be weak relative to others. This phenomenon is likely due to the lowest crystallite size presented by the sample [47]. Peaks labeled with asterisks (*) correspond to the SnO_2_ phase from the FTO substrate [48]. 

The Debye–Scherer relation in Equation (2) was used to determine the average crystallite sizes, where *D* is the crystallite size, *K* is a constant (0.9), λ is the wavelength of CuK_α_ X-rays equal to 0.15418 nm, β is the full-width at half-maximum (FWHM) of the preferential peak in radians, and θ is the Bragg angle [49].

(2) $$D=\frac{K\lambda }{\beta cos\theta }$$

**Figure 5:**
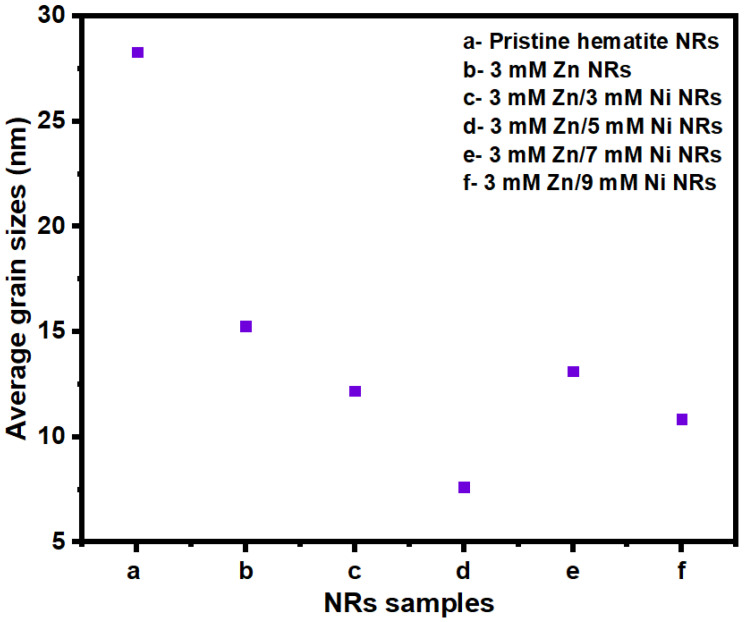
Crystal sizes for pristine hematite NRs, 3 mM Zn, 3 mM Zn/3 mM Ni, 3 mM Zn/5 Mm Ni, 3 mM Zn/7 mM Ni, and 3 mM Zn/9 mM Ni NR samples.

The crystallite sizes obtained were between 7.62 and 28.29 nm, as shown in Figure 5. The three most intense peaks of hematite, (104), (110), and (116), were used in the estimation of the crystal sizes of the films. The sizes generally decreased down to the value of 7.62 nm with increasing dopant concentration for the 3 mM Zn/5 mM Ni NRs sample. This observation is consistent with previous findings obtained for tin (Sn)-doped hematite films [43]. Studies conducted by Lassoued et al. (2018) further reveal that the decrease in crystallite sizes with the increase in Ni dopant concentration is a result of enhanced nucleation of particles in the host sample [50]. However, a further increase in Ni dopant concentration for 3 mM Zn/7 mM Ni NRs and 3 mM Zn/9 mM Ni NRs samples resulted in an increase in the crystal sizes relative to the value observed for 3 mM Zn/5 mM Ni NRs. This may be associated with the agglomeration of tiny crystallites due to the high Ni dopant concentration used in the film preparation [51]. 

### 3.3. Raman Characterization

**Figure 6:**
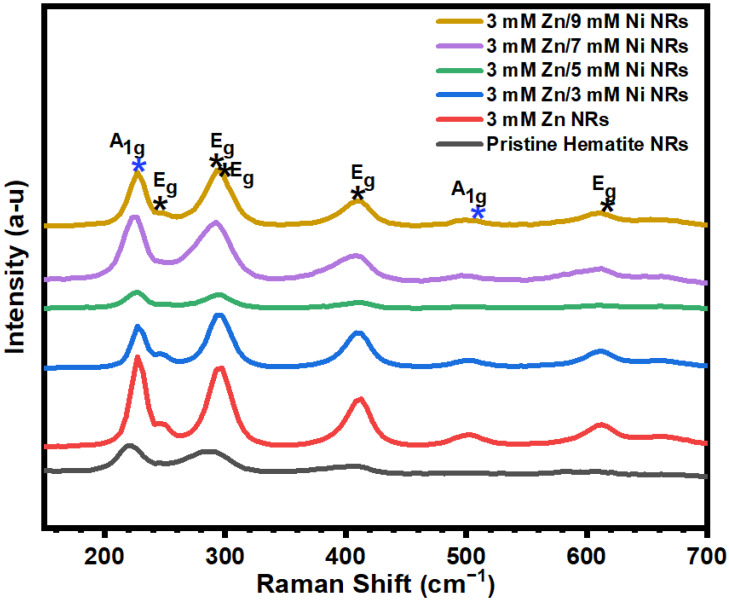
Raman spectra of pristine and doped hematite NRs grown over hematite seed layers.

Raman spectroscopy was carried out to further examine the effects of Zn and Ni dopants on the crystalline hematite phase. The Raman spectra of all the samples are presented in Figure 6. The seven Raman vibrational modes of hematite were observed at 225, 247, 293, 299, 412, 498, and 613 m cm^−1^ in all of the samples’ spectra. The A_1g_ vibrational modes are associated with the peaks at 225 and 498 cm^−1^. The five E_g_ modes observed for the samples correspond to the peaks at 247, 293, 299, 412, and 613 cm^−1^, respectively [52]. No additional vibrational modes other than those of hematite were observed. This further confirms the absence of impurities and the possibility of defects in the prepared NRs, in agreement with the XRD analysis of the films. A slight blue shift of the peaks was observed for the doped NRs samples compared to the pristine hematite NRs. This shift is similar to observations reported for copper (Cu)- [53] and chromium (Cr)-doped [54] hematite films and is attributed to the incorporation of the dopants in the hematite lattice. Initially, an increase in peak intensities was observed for the 3 mM Zn and 3 mM Zn/3 mM Ni NRs samples compared to the pristine hematite NRs. However, a remarkable reduction in the peak intensity of the 3 mM Zn/5 Mm Ni NR sample was observed relative to other samples. This is in agreement with reports that the Raman intensities are directly proportional to crystallite sizes [55,56]. Moreover, a similar trend of results was noted for the crystallite sizes of the doped NR samples. However, despite the pristine hematite sample presenting the largest crystallite size, its intensity was the second lowest after that of the 3 mM Zn/5 Mm Ni NRs. This is ascribed to adjustments in the hematite surface and grain boundary disorientations due to the surface passivation effect of the dopants of the doped NRs relative to that of the pristine hematite NRs [57]. Furthermore, a weak LO phonon mode was noticed at about 660 cm^−1^ for the doped hematite NRs. Based on previously reported results, this peak is likely attributed to surface defects or stress as a result of the dopants [54].

### 3.4. Elemental Composition

**Figure 7:**
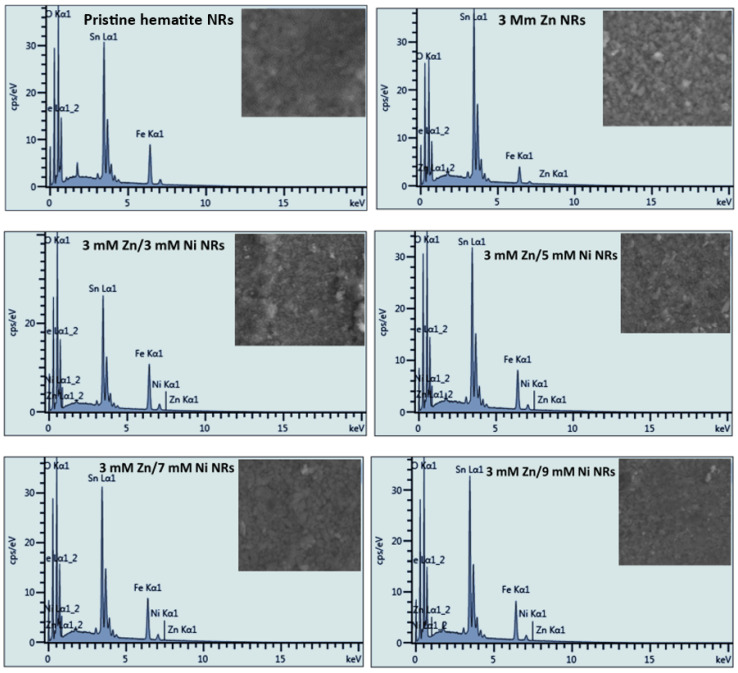
EDS analysis of pristine hematite and Zn- and Ni-doped NR samples.

EDS analysis of the undoped and doped hematite NRs samples was conducted to establish the composition of the different chemical elements in the samples. The chemical species of iron (Fe) and oxygen (O) for hematite and tin (Sn) from tin oxide (SnO_2_) on FTO substrates were observed for all of the NRs samples. The Fe and O k-line signals were observed at 6.42 and 0.53 keV, respectively, and correspond to previous findings [58]. The EDS results also show the Zn element in the 3 mM Zn NRs sample. In addition, Zn and Ni were detected in the spectra of all Zn/Ni co-doped NRs, as shown in Figure 7. 

Additionally, Table 1 shows the different atomic percentage compositions of the pristine and doped NR samples. Although the atomic composition of Zn was not uniform across the samples, that of Ni increased with the increase in concentration, similar to the stoichiometric increase in dopant concentrations used. No other elements were detected. This implies that the nickel and zinc dopants were successfully incorporated into the hematite NRs, and hence, it further validates the purity of the samples depicted by the Raman and XRD analysis of the films.

### 3.5. UV-Vis Absorption

**Figure 8:**
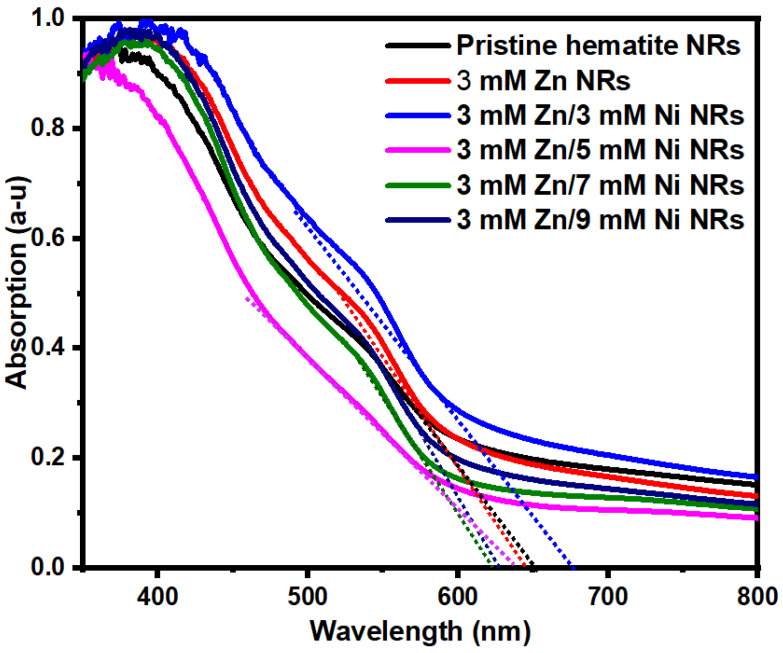
Absorption spectra of hematite NRs co-doped with different Zn/Ni concentrations.

The light absorption of the pristine hematite NRs and the 3 mM Zn, 3 mM Zn/3 mM Ni, 3 mM Zn/5 Mm Ni, 3 mM Zn/7 mM Ni, and 3 mM Zn/9 mM Ni doped NRs samples was investigated within a 350–800 nm wavelength range, and the results are shown in Figure 8. All absorption spectra show peaks at about 400 nm. This is consistent with the reported wavelength of the maximum photon absorption for hematite [59], assigned to Fe^3+^ d-d transitions [58,60]. The onset absorption wavelength for all samples was approximately 600 nm. The UV-vis absorption properties of NRs showed no direct correlation between the absorbance and the dopant concentrations. Nevertheless, the observed variations in photon absorption by the samples might be associated with differences in light scattering at the surface of the hematite NRs due to changes in their particle sizes, film thickness, and other intrinsic modifications resulting from Zn/Ni doping [61,62].

The bandgap values for the samples were estimated using the expression

(3) $$Eg\left(\mathrm{eV}\right)=\frac{1240}{\lambda \left(\mathrm{nm}\right)}$$

where *Eg* is the bandgap, 1240 is a constant obtained from the product of Planck’s constant (h = 4.14 × 10^−15^ eV) and the speed of light (c = 3 × 10^8^), and *λ* is the wavelength obtained from the intercept of the extrapolated linear section of the absorption spectrum on the wavelength axis [3]. The bandgaps obtained were 1.90, 1.92, 1.92, 1.95, 1.99, and 1.98 eV for pristine hematite, 3 mM Zn, 3 mM Zn/3 mM Ni, 3 mM Zn/5 Mm Ni, 3 mM Zn/7mM Ni, and 3 mM Zn/9 mM Ni NRs samples, respectively. The values obtained are within the bandgap range of 1.9–2.2 eV of hematite [63].

### 3.6. PEC Measurements

#### 3.6.1. LSV Measurements

**Figure 9:**
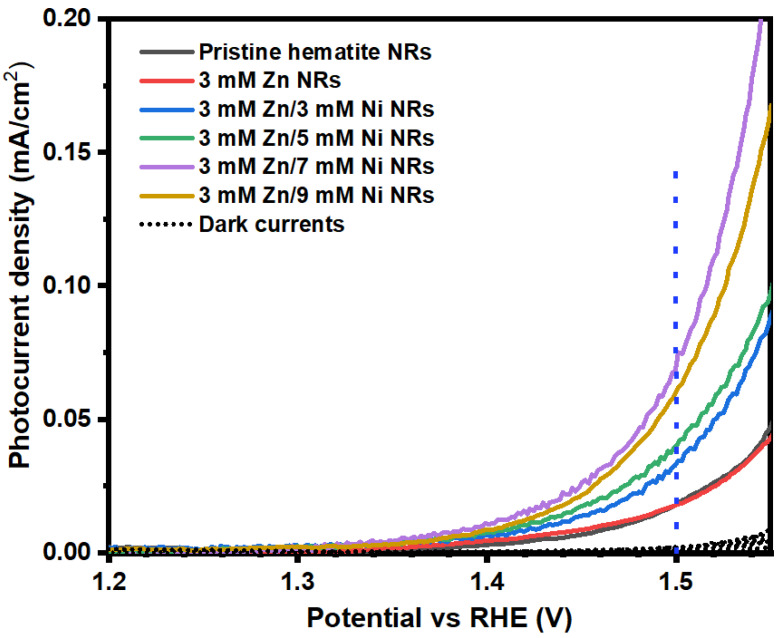
Photocurrent density plots of hematite NRs co-doped with different Zn/Ni concentrations.

To investigate the PEC performance of the pristine and Zn/Ni co-doped hematite NRs, LSV measurements were performed on the prepared samples in dark and light conditions, and the results are shown in Figure 9. The dark currents were notably small compared to the corresponding photocurrent density values under illumination. Upon irradiation, the pristine hematite exhibited a photocurrent of 0.019 mA/cm^2^ at 1.5 V vs. RHE. There was no significant difference in photocurrent densities observed for the 3 mM Zn-doped and pristine hematite NRs samples. This was likely due to the low Zn dopant concentration of 3 mM used in the preparation of the doped sample. Previous studies have also shown that the application of very low dopant volumes presents no significant effects on the properties of the host material [64]. The introduction of additional Ni dopant concentrations to the 3 mM Zn NRs samples yielded better photocurrent densities. The photocurrent densities increased with increasing Ni dopant concentration. The 3 mM Zn/7 mM Ni NRs sample presented the highest photocurrent of 0.072 mA/cm^2^ at 1.5 V vs. RHE. This photocurrent density was 279% higher when compared to the value observed for pristine hematite and 3 mM Zn-doped NRs films. This improvement could be due to enhanced surface reaction kinetics at the interface of 3 mM Zn/7 mM Ni NRs films with the electrolyte [38]. Moreover, related studies on hematite NRs films facilely doped with Ni and chromium (Cr) suggest that the incorporation of the dopants suppresses the recombination of electrons and holes by modifying the semiconductor interface to inhibit the density of surface states [50,65]. The photocurrent density, however, dropped with the 3 mM Zn/9 mM Ni NRs sample to 0.061 mA/cm^2^ at 1.5 V vs. RHE. This result revealed the occurrence of the maximum Ni dopant level for hematite NRs. For the 3 mM Zn/9 mM Ni NRs samples, the Ni dopant concentration exceeded the optimal level, which likely increased the recombination centers at the semiconductor–electrolyte interface, resulting in a drop in photocurrent density [65]. The 3 mM Zn/7 mM Ni NRs sample exhibited the lowest onset potential of 1.32 V vs. RHE, which is paramount in offsetting the energy barrier across the semiconductor–electrolyte interface for enhanced charge transport [66]. 

#### 3.6.2. Mott–Schottky Analysis

**Figure 10:**
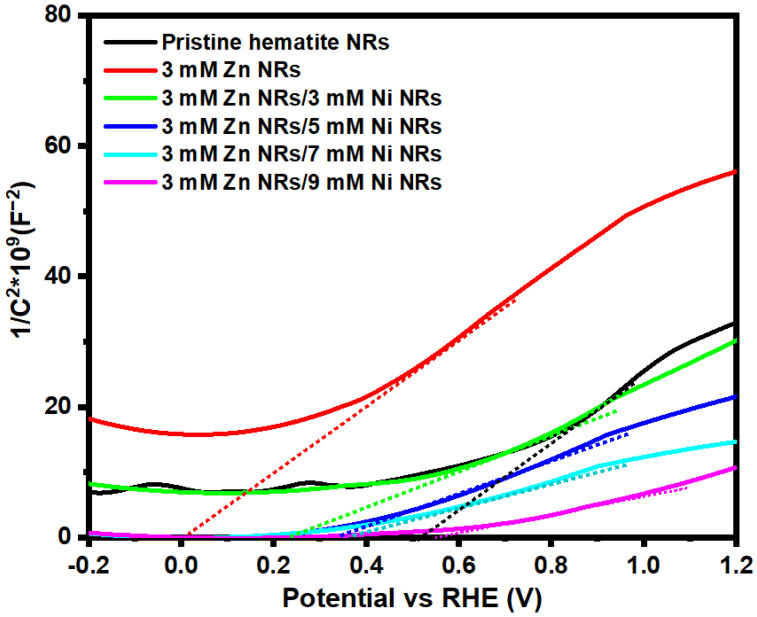
Mott–Schottky spectra of seeded hematite NRs co-doped with different Zn/Ni concentrations.

Figure 10 shows the Mott–Schottky plots of the as-prepared pristine and doped hematite NRs samples. The positive slopes of the plots confirmed the n-type nature of hematite [13,42]. Table 2 shows the flat band potential ($V_{fb}$) and the donor density ($N_{D}$) results obtained from the Mott–Schottky plots of the samples. The $V_{fb}$ values were derived from the x-axis intercepts of the extrapolated linear sections of the plots, whereas the $N_{D}$ values were worked out from the slopes of the plots, in line with the Mott–Schottky relation given in Equation (4):

(4) $$C^{-2}=\frac{2}{e\varepsilon \varepsilon_{0}A^{2}N_{D}}\left(V-V_{fb}-\frac{kT}{e}\right)$$

where C is the space charge capacitance, *e* is the electron charge, ε is the dielectric constant (ε=80 for hematite), $\varepsilon_{0}$ is the permittivity of free space, A represents the surface area of the electrode, $N_{D}$ is the donor density, V is applied voltage, $E_{fb}$ is flat band potential, k is the Boltzmann constant, and *T* is the temperature in kelvin [38].

The flat band potential is a key parameter for PEC semiconductor electrode materials since they reflect the hole transport mechanism occurring at the electrode surface region [67]. As a baseline sample, the pristine hematite NRs presented a $V_{fb}$ of −0.43 V vs. RHE. An increase of 0.41 V was realized in $V_{fb}$ when the Zn dopant was introduced for the 3 mM Zn NR sample. This is quite contrary to other reports [26,68] and might be attributed to the low dopant concentration used. However, a cathodic shift in $V_{fb}$ values was observed with increasing Ni dopant concentration. The 3 mM Zn/9 mM Ni and 3 mM Zn/7 mM Ni NRs produced the lowest and second lowest $V_{fb}$ values of −0.70 and −0.65 V vs. RHE, respectively. According to Kumari et. al., this could be linked to enhanced hole transport at the NR–electrolyte interface during oxygen evolution [26]. 

The $N_{D}$ values of the pristine hematite NRs, 3 mM Zn, 3 mM Zn/3 mM Ni, 3 mM Zn/5 Mm Ni, 3 mM Zn/7 mM Ni, and 3 mM Zn/9 mM Ni NRs samples are shown in Table 1. All $N_{D}$ values of the doped samples are within the same order of magnitude of 10^19^ cm^−3^. The initial introduction of the Zn dopant for the 3 mM Zn NR sample lowered the $N_{D}$ of the NRs by 22.5% compared to 1.38 × 10^19^ (cm^−3^) obtained for the pristine hematite NRs, which is consistent with observations by Kumari et al. [69]. While Zn dopants are responsible for enhancing charge separation in the bulk and interface of the hematite photoanode, the reduction in $N_{D}$ is attributed to its low water oxidation potential as a p-type element [70]. Additionally, the results show that an increase in the nickel dopant concentration of the Zn-doped samples significantly increased the $N_{D}$ values across the hematite NRs samples. The maximum $N_{D}$ value of 2.89 × 10^19^ (cm^−3^) was obtained for the 3 mM Zn/7 mM Ni NR sample, which was 2.1 times higher than that of the pristine hematite NRs. This observation indicates that the varied Ni dopant concentrations were well integrated into hematite as ionized donors and likely established energy traps within the films [71]. The increase in $N_{D}$ values will directly improve the conductivity of the co-doped samples and their PEC activity [72]. This is the key reason for the maximum photocurrent density obtained for the 3 mM Zn/7 mM Ni co-doped films. Furthermore, increasing the dopant concentration within optimized proportions has been reported to increase the photocurrent density of hematite films [41]. However, a 7% reduction in $N_{D}$ was observed for the 3 mM Zn/9 mM Ni NRs compared to that of 3 mM Zn/7 mM Ni NRs. Higher dopant concentrations are associated with a greater tendency for the dopant ions to bind to oxygen, leading to the formation of metal oxides [73]. This phenomenon could have led to the decrease in the $N_{D}$ value for the 3 mM Zn/9 mM Ni NRs, since metal oxides hinder the deep penetration of dopants into the bulk of the film. A similar trend toward a slight reduction in $N_{D}$ with increasing Ni doping concentration has previously been reported for hematite films and was attributed to the possible formation of a blocking layer by the dopant, consequently limiting their light absorption and PEC activity [44]. 

**Figure 11:**
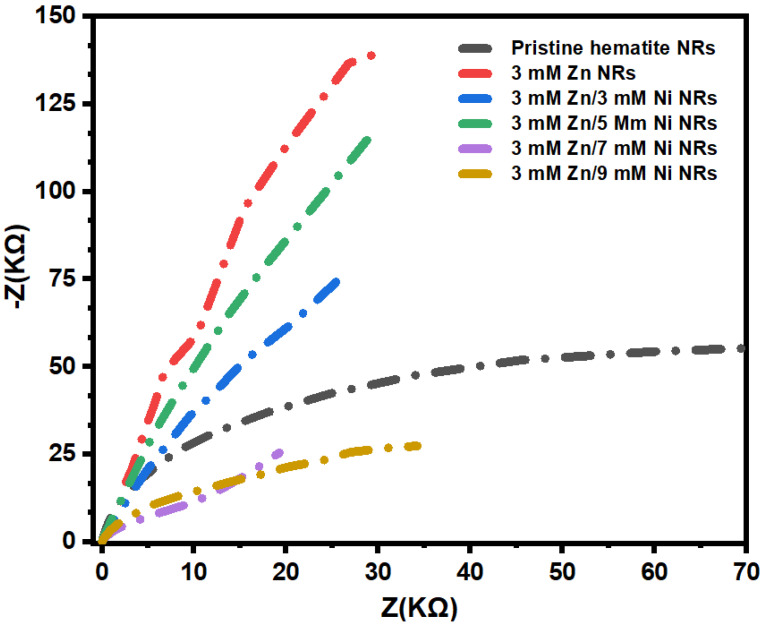
Electrochemical impedance Nyquist plots of pristine, Zn-doped, and Zn/Ni-doped hematite NR photoanodes.

To further examine the Zn/Ni dopant effect on the charge mobility dynamics at the surface interface of the hematite NRs films, electrochemical impedance measurements were conducted. The plots obtained from the as-prepared photoanodes are shown in Figure 11. The 3 mM Zn/7 mM Ni NRs exhibited the smallest diameter, indicative of the lowest resistance to charge transport for this sample. This further confirms the highest photocurrent density for the 3 mM Zn/7 mM Ni NRs photoanode [3]. The pristine hematite NRs revealed the largest path, in agreement with previous observations for typical n-type semiconductor photoanodes in PEC setups [74]. The largest path is associated with the highest resistance to the transfer of holes to the semiconductor–electrolyte interface for oxidation. Generally, the Zn/Ni co-dopant proportions enhanced the charge transfer at the hematite–electrolyte interface. 

## 4. Conclusions

Hematite NRs were hydrothermally synthesized over spin-coated seed layers and co-doped with a constant concentration of 3 mM Zn and varied concentrations of 0, 3, 5, 7, and 9 mM Ni. The doped samples were annealed at 550 °C for 1 h. The samples presented a largely uniform morphology of vertically aligned NRs with slight inclinations. There was no significant effect of the facile Zn/Ni co-dopants on the surface morphologies of the NRs. The XRD analysis revealed a hematite crystalline phase of good purity for all samples, which was further confirmed by the Raman spectra of the films. The photon absorption of the pristine and doped NRs samples was high within the visible range of the electromagnetic spectrum and is related to their estimated bandgap range of 1.9–2.2 eV. The 3 mM Zn/7 mM Ni NRs co-doped sample exhibited the highest PEC performance with a photocurrent density of 0.072 mA/cm^2^ at 1.5 V vs. RHE. The high *N_D_* value estimated for the 3 mM Zn/7 mM Ni NRs sample was the key reason for the improved photocatalytic activity observed for the films. Overall, this study presents a new approach to enhancing the PEC activity of hematite NRs by facile co-doping with Zn and Ni. 

## Figures and Tables

**Table 1:** Chemical atomic composition (at %) of the pristine and doped hematite NRs.

NR Samples	Element (at%)				
	O	Fe	Sn	Zn	Ni
Pristine hematite NRs	70.90	14.00	15.10	-	-
3 mM Zn NRs	70.75	13.26	15.94	0.05	-
3 Mm Zn/3 Mm Ni NRs	69.76	17.23	12.93	0.04	0.04
3 Mm Zn/5 Mm Ni NRs	70.18	13.12	16.49	0.10	0.11
3 Mm Zn/7 Mm Ni NRs	69.87	14.09	15.78	0.07	0.19
3 Mm Zn/3 Mm Ni NRs	70.50	12.60	16.58	0.04	0.28

**Table 2:** Flat band potential ($V_{fb}$)/V and donor densities $N_{D}$ × 10^19^/cm^−3^ of pristine and doped hematite NR samples synthesized over hematite seed layers.

**NR Sample**	$V_{fb}$ **(V)**	$N_{D}$ **× 10^19^ (cm^−3^)**
Pristine hematite NRs	−0.43	1.38
3 mM Zn NRs	−0.02	1.07
3 Mm Zn/3 Mm Ni NRs	−0.36	1.55
3 mM Zn/5 mM Ni NRs	−0.38	2.01
3 mM Zn/7 mM Ni NRs	−0.65	2.89
3 mM Zn/9 mM Ni NRs	−0.70	2.69

## Data Availability

The data generated and or analyzed in this work can be obtained from the corresponding author upon reasonable request.
